# Driving time drives the hospital choice: choice models for pelvic organ prolapse surgery in Italy

**DOI:** 10.1007/s10198-022-01563-6

**Published:** 2023-01-11

**Authors:** Amerigo Ferrari, Chiara Seghieri, Andrea Giannini, Paolo Mannella, Tommaso Simoncini, Milena Vainieri

**Affiliations:** 1 Institute of Management, MeS (Management and Health) Laboratory, Sant’Anna School of Advanced Studies, Via San Zeno 2, 56127 Pisa, Italy; 2 Department of Clinical and Experimental Medicine, Division of Obstetrics and Gynaecology, University of Pisa, Via Roma 67, 56126 Pisa, Italy

**Keywords:** Pelvic organ prolapse, Gynecological surgery, Hospital choice, Patient mobility, Discrete choice analysis, Mixed logit model, I12

## Abstract

**Objective:**

The Italian healthcare jurisdiction promotes patient mobility, which is a major determinant of practice variation, thus being related to the equity of access to health services. We aimed to explore how travel times, waiting times, and other efficiency- and quality-related hospital attributes influenced the hospital choice of women needing pelvic organ prolapse (POP) surgery in Tuscany, Italy.

**Methods:**

We obtained the study population from Hospital Discharge Records. We duplicated individual observations (*n* = 2533) for the number of Tuscan hospitals that provided more than 30 POP interventions from 2017 to 2019 (*n* = 22) and merged them with the hospitals’ list. We generated the dichotomous variable “hospital choice” assuming the value one when hospitals where patients underwent surgery coincided with one of the 22 hospitals. We performed mixed logit models to explore between-hospital patient choice, gradually adding the women’s features as interactions.

**Results:**

Patient choice was influenced by travel more than waiting times. A general preference for hospitals delivering higher volumes of interventions emerged. Interaction analyses showed that poorly educated women were less likely to choose distant hospitals and hospitals providing greater volumes of interventions compared to their counterpart. Women with multiple comorbidities more frequently chose hospitals with shorter average length of stay.

**Conclusion:**

Travel times were the main determinants of hospital choice. Other quality- and efficiency-related hospital attributes influenced hospital choice as well. However, the effect depended on the socioeconomic and clinical background of women. Managers and policymakers should consider these findings to understand how women behave in choosing providers and thus mitigate equity gaps.

**Supplementary Information:**

The online version contains supplementary material available at 10.1007/s10198-022-01563-6.

## Introduction

### Theoretical background

The Italian healthcare jurisdiction promotes patient choice as well as patient mobility across different Local Health Authorities and Regions [1, 2]. Patient choice has been fostered by market-based health policies and reforms aimed at enhancing competition among different providers to promote the performance improvement [3, 4]. As a matter of fact, hospitals receive a price (or reimbursement) from the Regional Health Authorities or the Ministry of Health, which is set according to the treatment received by patients. Therefore, patient choice becomes crucial to incentivize competition among providers to improve hospital care, and thus attract users and revenue [5]. Similar reforms have been introduced in several high-income countries, such as the UK, Sweden, and France to increase the efficiency and quality of the provided health services and empower the patient role in the decision-making process [6].

In such frameworks, where competition among providers exists and patients can access any provider, the choice of where to be visited and treated is held by patients [7]. Patient choice depends on several heterogeneous inputs, such as service quality, efficiency and reputation, availability of facilities and technologies, recommendations, prior experiences, and attitudes and behaviors of health professionals [8–11]. Other accessibility-related factors such as costs, physical proximity, acceptability, and adequacy of service supply in relation to the population may also influence patient choice [12, 13]. Although the choice is usually influenced by the physician’s advice, patients tend to choose the highest quality provider, or the nearest, or the fastest available [14, 15]. Among all these determinants, patient mobility—intended as willingness to travel—may be of major interest to assess service capacity and determine how freely patients access health services [16, 17]. Indeed, the proximity to the place of residence has been identified as a key determinant of patient choice, with a variable effect depending on the patient’s socioeconomic background [18, 19].

There is a large literature on between-hospital patient choice using patient-episode level administrative data to explore the effect of distance and reputation of hospitals on patient demand [5, 11, 20–22]. Our work explores the determinants of between-hospital patient choice for pelvic organ prolapse (POP) surgery in Tuscany, Italy. We selected POP because it is a benign disease requiring elective planned surgery, for which women have the chance to choose—contrary to emergency conditions. Moreover, POP is a common condition among women, affecting up to 40% of the female population with a 7–11% lifetime surgical risk [23, 24]. Data from the Performance Evaluation System of Sant’Anna School of Advanced Studies shows that high volumes of POP interventions are delivered every year in Tuscany (989 in 2018) [25–27]. Finally, the last report by the Regional Health Agency of Tuscany reveals that 90 per 100,000 women underwent POP surgery in 2014 [28].

### Overview of the clinical condition

Pelvic organ prolapse (POP), defined as the downward descent of pelvic organs towards the vagina, is a pelvic floor disorder occurring and deteriorating over time after menopause, driven by the endocrine modifications encountered with menopause and by the aging process. POP prevalence is constantly growing because of the increase in life expectancy. It negatively impacts on social and psychophysical functionality, also because it is usually associated to urinary/fecal incontinence or obstruction, thus leading to severe limitations to daily independence and social interactions. While POP is not a life-threatening condition, it does not have a self-limited course, and often women receiving treatment are those who have the possibility to access health services, travel, and pay [29–31].

The choice of the surgical procedure depends on various cultural and clinical factors, such as surgeon’s expertise, resource availability, and patient preferences. Particularly, POP surgery can be performed vaginally or through an abdominal approach, which can be either open traditional laparotomy or minimally invasive surgery. Several meta-analyses have demonstrated the superiority of the minimally invasive abdominal approach over the laparotomic approach in terms of complications, blood loss, and length of stay [32, 33]. Particularly, minimally invasive surgery consists of reconstructive techniques employing an abdominal access with the use of a small incision and a video laparoscopic tool: it can be performed either laparoscopically or robotically [34].

### Possible contribution of this work to the matter of practice variation

Since POP surgery is a type of elective surgery prone to practice variation and large differences in treatment rates among Tuscan health districts have already been documented [28, 35], this article can partly contribute to understanding the determinants of such variation. Indeed, practice variation is a major topic in elective surgery, as being related to the equity of access to health services, which is one of the threefold missions of healthcare systems pursuing universal coverage [36, 37]. As variation should be driven just by patients’ preferences, unwarranted variation—not justified by real differences in patients’ needs—should be reduced [38, 39]. However, to define the observed variation as unwarranted, all determinants of patients’ choices should be analyzed [40]. Besides, understanding which factors hinder or facilitate patient choice allows to efficiently support resource allocation without affecting the quality and efficiency of services delivered to the population [41].

Therefore, this paper aims to explore how travel times, waiting times, and other quality- and efficiency-related hospital attributes influence the hospital choice of patients undergoing POP surgery in Tuscany, considering at the same time the patients' demographics to incorporate equity expectations. In so doing, this work may reveal the existence of practice variation and suggest some factors that may partly explain it, thus providing policymakers and health managers with new evidence on which they can act to reduce unwarranted variation, pursuing the goal of ensuring more uniform and equitable access to urogynecological surgery services according to the principles of universal health coverage.

## Methods

### Setting and study design

The Italian National Health Service ensures free universal coverage following a decentralized model. The central government is responsible for setting the overall funding requirements and goals and ensuring equity of care across its territory, while Regions organize and provide the healthcare services within their territories, with a high degree of administrative, political, legislative, and fiscal autonomy [1]. Among the Italian Regional Health Services, Tuscany is a large Region in Central Italy responsible for the healthcare services provided to the 3.7 million inhabitants, receiving around 6% of the healthcare fund. Tuscany is divided into 3 Local Health Authorities, 4 Teaching Hospitals (of which one is only pediatric), and 26 health districts, financed by a capitation-weighted budget, with more than 95% of hospitals being public. The last assessment by the Italian Ministry of Health shows that Tuscany is one of the most performing Italian Region.

Similar to other Italian Regions, Tuscany has a non-competitive Health Service where patients are free to choose the provider. Studies on patient choice can either employ data from survey, where individuals are asked to choose between different hypothetical scenarios or to report about recent health-related episodes (stated preferences) [42], or administrative data, which allow to retrospectively analyze the choice of patients accessing health services (revealed preferences) [21]. Particularly, this paper used anonymized individual-level administrative health data (namely, Regional Hospital Discharge Records) to examine the relationship between travel and waiting times, as well as hospital efficiency and quality, and the hospital choice of patients who received POP surgery in Tuscany from 2017 to 2019. As a retrospective observational study, it has been reported according to the STROBE guidelines.

### Data source

Our research laboratory can access regional administrative health data thanks to a collaboration agreement with the Regional Health Service of Tuscany, which routinely shares regional health databases with our research laboratory. Tuscany also funds our research activities despite having no role in formulating the research questions, choosing the study design, collecting and analyzing data, or writing and submitting the manuscript for publication.

We used Hospital Discharge Records, which include information on the diagnosis and treatment received by each patient, together with sociodemographic and residence data. The Regional Health Information Office routinely checks data quality and ensures anonymization by assigning each patient with an encrypted unique identifier equal in all administrative databases. Thanks to this identifier, the patient’s identity and other sensitive information are unknown. The study complies with the Italian law on privacy 101/2018 (aligned with the European GDPR 2016/679); therefore, according to the Italian Data Protection Authority, neither ethical approval nor informed consent was necessary [43].

### Population and hospitals

The analysis included all women over 40 years and residing in Tuscany who had a planned hospitalization for receiving POP surgery from 2017 to 2019. Data after 2019 were excluded since the Covid-19 pandemic resulted in interruptions and delays in the delivery of non-emergency health services from March 2020 and in restrictions on patients’ free choice, which may have compromised the interpretation of the results. Patient selection was performed on SAS Software using the appropriate ICD-9-CM (International Classification of Diseases, 9th Revision) codes, as shown in Table S4. These codes were validated with hospital gynecologists who employ them for administrative reasons.

Moreover, patients receiving just transvaginal anterior/posterior colporrhaphy with no concomitant hysterectomy were excluded (Table S4), as we sought to focus on major surgical interventions with similar indications and outcomes performed for advanced apical or multicompartmental POP for which a reconstructive approach is needed. Therefore, just women undergoing abdominal surgery or transvaginal surgery with concomitant hysterectomy were selected. Patients diagnosed with cancer or trauma and patients in the major diagnosis category of pregnancy were also excluded. Despite having no information on the specific surgical technique performed on each woman (e.g., sacrocolpopexy), ICD-9-CM codes were used to identify the surgical approach (Table S4).

We identified 2819 women who received POP surgery during the study period and calculated surgical treatment rates in each health district of Tuscany. Furthermore, by comparing the variables “health district of residence” and “health district of provision” available in our database, we defined patient mobility as the percentage of women residing in a certain health district who received surgery in a health district other than their district of residence. We then attempted to correlate at the health district level mobility with treatment rates by calculating Pearson’s correlation coefficient.

Among these 2819 women, we selected those 2556 patients that had surgery in Tuscan public hospitals that provided more than 30 POP interventions during the years of analysis (*n* = 22). Then, we calculated for each patient the travel time she would spend to reach any of the 22 hospitals included in the study from the centroid of her place of residence. Unfortunately, information on the place of residence was missing for 23 out of 2556 women. Therefore, our final study population was reduced to 2533 women. Travel distances were obtained from the regional road network, available on the Open Toscana website (http://open.toscana.it/). For this purpose, each of the 22 hospitals was integrated into a GIS environment and geolocated over the 26 health districts of Tuscany, as previously shown by our research group [44]. The process for estimating travel distances is described in Table 1.

**Table 1:** Characteristics of women and hospitals

Women (*n* = 2533)	
Age, mean (± SD)	67.6 (± 9.2) years
Age class, % (*n*)	
40–60 years	18.7 (473)
60–80 years	72.7 (1842)
> 80 years	8.6 (218)
Citizenship, % (*n*)	
Italian	95.5 (2418)
Non-Italian	4.5 (115)
Educational level, % (*n*)	
Low education (elementary or middle school)	69.1 (1469)
High education (high school or university)	30.9 (657)
Missing	407
Elixhauser comorbidity index, % (*n*)	
0	95.9 (2429)
1	3.35 (85)
2	0.7 (18)
3	0.05 (1)
Travel time^a^	
Mean (± SD)	19.1 (± 16.6) minutes
Median (IQR)	14.7 (7.3–25.6) minutes
Hospitals (*n* = 22)	
Annual median waiting times, mean (± SD)	148.8 (± 80.6) days
Annual volumes of POP interventions, mean (± SD)	39.3 (± 26.6)
Annual average length of stay, mean (± SD)	3.5 (± 0.8) days

Women (*n* = 2533) were characterized by nationality (Italian *vs.* non-Italian), age class (40–60 *vs.* 60–80 *vs.* > 80 years), and educational level used as a proxy of the economic status (elementary or middle school *vs.* high-school or university). We also computed for each patient the Elixhauser Comorbidity Index [45] using the approach described by Van Walraven et al*.* (2009) suitable for administrative data [46].

On the other hand, we characterized each hospital (*n* = 22) according to three quality- and efficiency-related features. We computed these hospitals’ characteristics (waiting times, volumes, length of stay) by referring them to the previous year than the one analyzed (thus in the period 2016–2018), assuming that hospital choice responded to the quality and efficiency indicators of the past year, as described by Gutacker et al. [47].

First, after computing for all women waiting times from the day when they booked the hospitalization for POP surgery to the day of hospital admission, we calculated median waiting times for each hospital. We used the median instead of the mean to reduce measurement and coding errors leading to outlier values. According to the literature, waiting times, rather than being influenced by hospital volume and capacity, depend on a combination of factors such as insufficient personnel, prescribing inappropriateness, and overall system inefficiency [17].

Second, to assess the hospitals’ efficiency [48], we computed the average length of stay (by removing outliers) after POP interventions for each hospital, as information on the hospital stay for each patient was available in regional databases. In fact, length of stay is one of the most frequently evaluated outcomes to investigate the efficiency of surgical care pathways, being closely related to costs and depending on the type of intervention [49].

Finally, to assess the hospitals’ quality, we calculated for each hospital the overall volumes of POP interventions delivered in 2016–2018. Although previous studies have pointed out some limitations in using volumes as indicators of quality [50, 51], several papers have demonstrated the association between higher volumes and better outcomes, making it possible to use volumes as a proxy for hospital quality in the absence of other more specific indicators [50, 52, 53].

### Hospital choice

We duplicated individual observations (*n* = 2533) for the number of Tuscan hospitals included (*n* = 22) and merged them with a list of hospitals including their features referred to the year prior to when each woman had surgery. We generated a dichotomous variable *hospital choice* assuming the value one when the hospital where the patient was operated on coincided with one of the 22 hospitals. Otherwise, the value was zero [21]. Between-hospital patient choice was explored through mixed logit models, which properly allow to account for heterogeneity in preferences by enabling coefficients to vary between patients and relaxing the assumption of independence from irrelevant alternatives [22].

More particularly, mixed logit models allow specifying dependent variables that have random coefficients, i.e., coefficients that vary randomly for each combination of patient and hospital. Hence, in our model, we have specified that the coefficient of travel times was unique—and, therefore, random—for each combination of patient and hospital created through the duplication process since travel times were calculated at the individual level. In contrast, the coefficients of hospital-level attributes (waiting times, volumes, length of stay) were the same for all patient replicates since such attributes were calculated at the hospital level. Please, see Table S5 to better understand how the database was constructed, and the caption of Table 2 for further details on the model selection and validation process.

**Table 2:** Results of mixed logit models

Mixed logit regression models (55,726 observations)						
	Model 1		Model 2		Model 3	
	Coeff.	SE	Coeff.	SE	Coeff.	SE
Main effects						
Waiting times	− 0.003***	0.001	− 0.001	0.001	− 0.003	0.003
Ln (travel times)	− 2.835***	0.068	− 2.891***	0.072	− 1.921***	0.340
Hospital attributes						
Total number of interventions			0.018***	0.001	0.017*	0.008
Average length of stay			0.016	0.036	− 0.042	0.311
Interactions with patient features						
Waiting times						
x Low education					− 0.001	0.001
x Elixhauser					0.004	0.002
x Age class					− 0.001	0.001
x Nationality					− 0.001	0.002
Ln (travel times)						
x Low education					− 0.717***	0.109
x Elixhauser					− 0.443	0.278
x Age class					− 0.107	0.101
x Nationality					− 0.405	0.288
Total number of interventions						
x Low education					− 0.005*	0.002
x Elixhauser					0.005	0.005
x Age class					− 0.001	0.002
x Nationality					− 0.008	0.006
Average length of stay						
x Low education					− 0.024	0.087
x Elixhauser					− 0.893**	0.286
x Age class					0.083	0.083
x Nationality					0.254	0.208
Standard deviation of individual heterogeneity						
Ln (travel times)	1.040***	0.058	1.111***	0.061	1.112***	0.075
Hospital Fixed Effects	No		No		Yes	
Log-likelihood	− 3854.9		− 3635.9		− 2893.8	

Bold values represent statistically significant coefficients

The model was performed on Stata Software using the “mixlogit” program. The cluster-robust standard error option was specified to account for clustering at the provider level; 50 Halton draws were used for the simulation, as appropriate

The coefficients represent the mean relative utility of each attribute conditional on the other attribute, whereas the standard deviation of random coefficients reflects the degree of heterogeneity among patients in the utility of the given attribute

Significance levels: *p < .05; **p < .01; ***p < .001

We built three models. Model 1 only included the main effects—waiting times (computed at the hospital level) and travel times (computed at the individual level). Travel times were added into the models as natural logarithms and were considered to have random coefficients varying among patients for each combination with the 22 hospitals. Model 2 also incorporated the other two quality- and efficiency-related hospital characteristics (total numbers of interventions and average length of stay).

To account for additional characteristics that could considerably influence women’s preferences and control for unobserved time-invariant heterogeneity across hospitals, we included Hospital Fixed Effects in Model 3, which absorb potential differences between hospitals that are persistent during the study period [54]. In addition, the women’s sociodemographic features were gradually added one-by-one to Model 3 as interactions with hospital attributes to explore how the sociodemographic background modified the effect of travel times, waiting times, and the other hospital attributes considered [21].

Finally, we estimated the effect of a change in travel and waiting times (main effects) on patient choice by computing the elasticity of demand at the individual level. Elasticity calculation was performed according to the elasticity model proposed by Sivey et al. [22]. Elasticity corresponds to the percentage change in demand associated with a 1% change in travel/waiting times and is expressed as mean elasticity of demand averaged across all Tuscan hospitals.

## Results

**Fig. 1:**
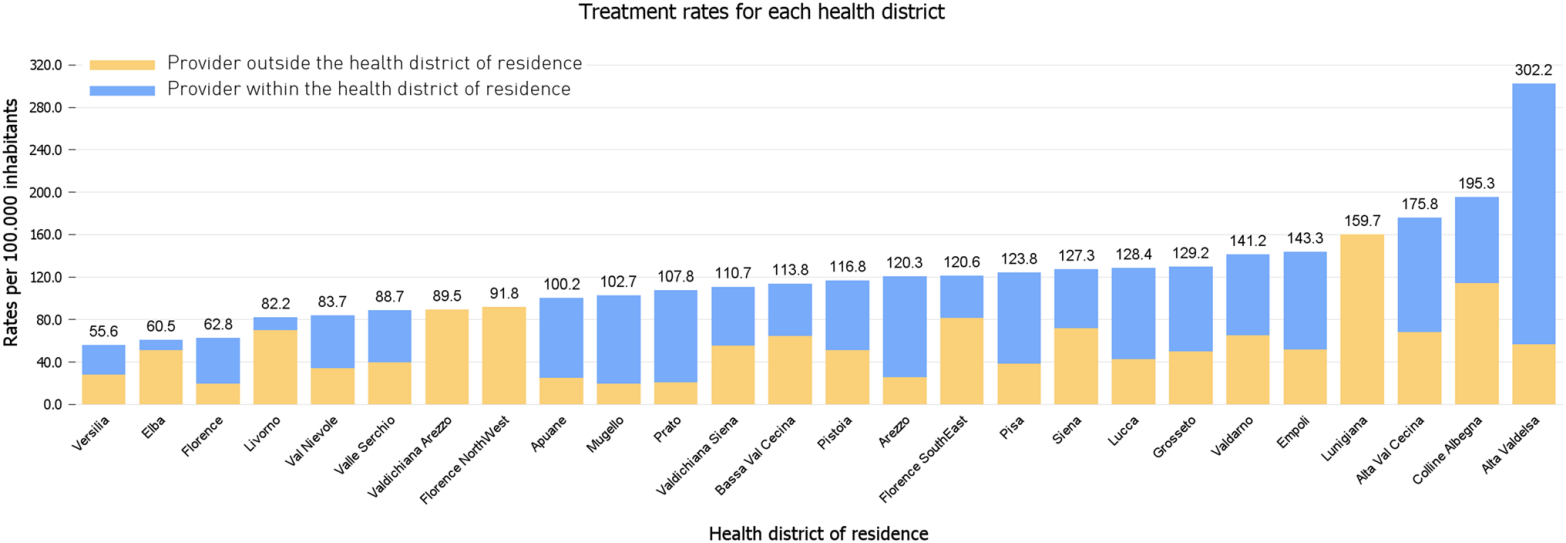
Three-year treatment rates for POP surgery in each health district of Tuscany from 2017 to 2019 (per 100,000 women). Treatment rates were computed by dividing the number of POP interventions for the 2017–19 female population residing in each health district regardless of where the intervention was provided. Total treatment rates were also split based on whether the provider hospital was in the same health district where each woman resided or in a different one. This information was obtained by comparing the two variables “health district of residence” and “health district of provision”. To visually observe patient mobility on the geographical map of Tuscany, please see Fig. 2

During the study period, 2819 women residing in Tuscany received POP surgery. Considering the female population over 40 years and residing in Tuscany from 2017 to 2019—which was obtained from the Aggregated Population Flow of Tuscany—, we computed treatment rates at the regional level and for each health districts, considering at the same time patient mobility across health districts and the type of intervention (Fig. 1 and Fig S3). Overall, 112.6 per 100,000 women underwent POP surgery in the 3-year period 2017–2019. However, treatment rates vary widely among health districts, as shown by the 5.4-fold variation between the lowest-rate and the highest rate districts (from 56 to 302 per 100,000).

**Fig. 2:**
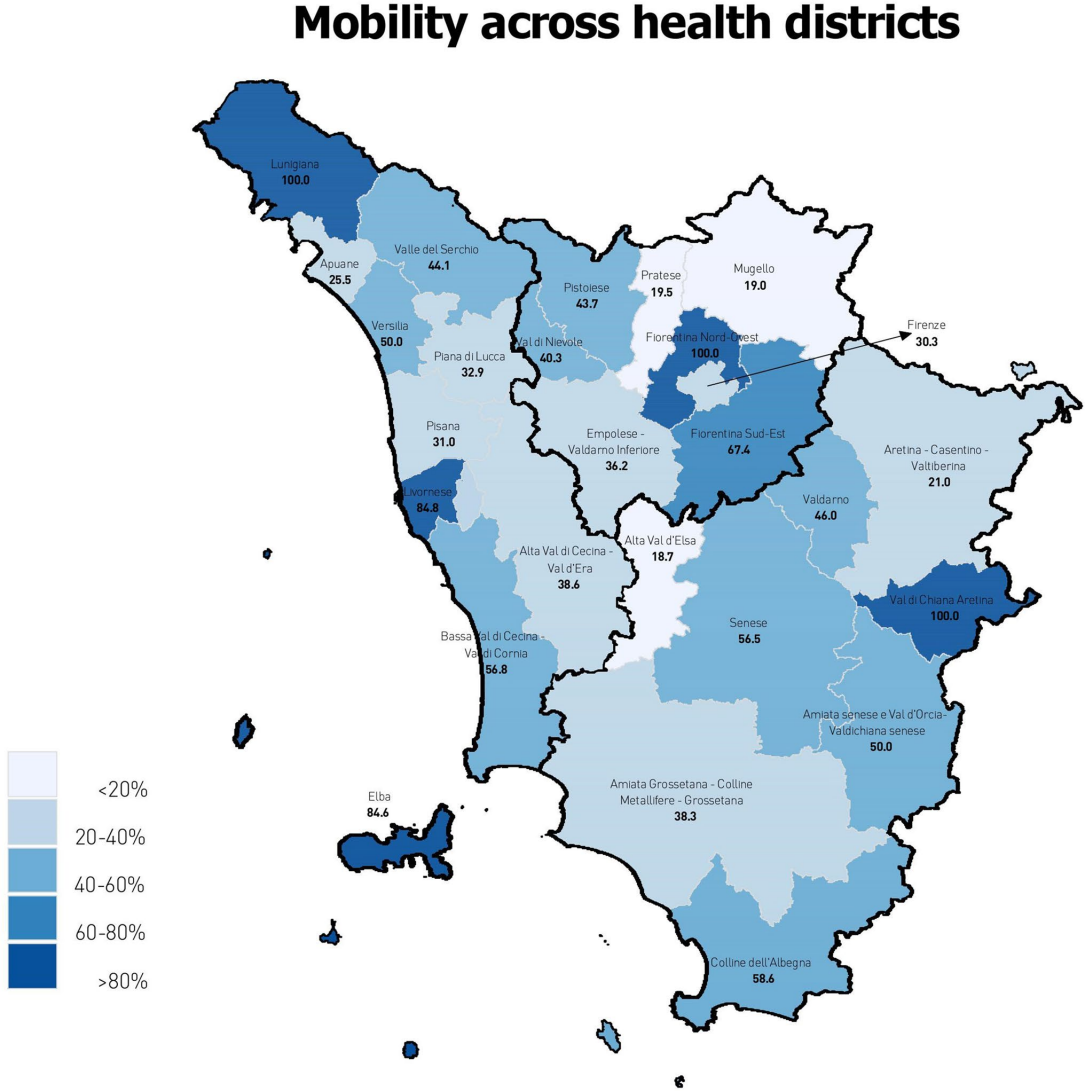
Mobility for POP surgery across the health districts of the Tuscany Region, expressed as percentage of POP interventions delivered outside the health district of residence

Comparing the variables “health district of residence” and “health district of provision”, total treatment rates in each health district were split according to where the provider hospital was located (Fig. 1). Six of the 26 health districts of Tuscany lacked a provider hospital within their territory (Florence North-West, Lunigiana, Valdichiana Arezzo) or had a provider hospital that delivered less than 30 interventions during the study period thus being excluded in following analyses (Elba, Livorno, Valle del Serchio). Figure 2 confirms this evidence, showing that from 2017 to 2019, there was 100% mobility from health districts lacking provider hospitals (Florence North-West, Lunigiana, Valdichiana Arezzo) to other districts. In addition, mobility from the Elba and Livorno districts, which had a provider hospital delivering less than 30 interventions, was about 85%. Except for the Lunigiana district, treatment rates in the other four districts with high mobility percentages were below the regional average (112.6), being among the ten lowest in Tuscany (Fig. 1 and Fig S3). Pearson’s coefficient showed an indirect correlation between mobility and treatment rates, although this association was rather weak (− 0.25).

### Population and hospitals

Among the 2819 residing women that received POP surgery, 2556 women were operated on in the 22 public Tuscan hospitals that provided more than 30 interventions during the study period. As information on the place residence was missing for 23 women (0.9% of the total), our final study population included 2533 patients. To perform regression analyses, the women’s data were duplicated for the number of Tuscan hospitals (*n* = 22); therefore, we obtained 55,726 observations.

As shown in Table 1, the mean age of our patients was 67.6 (± 9.2) years, with 73% of women aged between 60 and 80 years. Most of them (95%) were Italian, and 69% of them had a low education. Most women (96%) had no comorbidities. Travel distances were expressed in time (minutes) and incorporated in the statistical models as natural logarithms to normalize data distribution. The mean travel time from the place of residence to the chosen hospital was 19.1 (± 16.6) minutes.

As for the hospitals’ characteristics (Table 1), the annual median waiting times were, on average, 148.8 (± 80.6) days, while the annual average length of stay was 3.5 (± 0.8) days. Finally, the annual volumes of POP interventions provided by the 22 hospitals that delivered more than 30 interventions during the study period were, on average, 39.3 (± 26.6).

### Hospital choice

As emerged from Model 1, the main effects were statistically significant (Table 2). The negative sign of the coefficients means that patient choice was negatively and significantly influenced by longer waiting (*p* < 0.001) and travel times (*p* < 0.001). Therefore, women preferred hospitals nearby and with shorter waiting times, even though the effect of travel times was greater than the effect of waiting times. Furthermore, there was a significant preference heterogeneity for lognormally distributed travel times (*p* < 0.001). After the inclusion of hospital attributes (Model 2), the coefficient of travel times remained negative and statistically significant (*p* < 0.001), while waiting times lost significance. Model 2 also showed that patients chose hospitals that provided higher numbers of total interventions (*p* < 0.001). There was once again a significant preference heterogeneity for travel times (*p* < 0.001).

In Model 3, which also included Hospital Fixed Effects, interaction between educational level and travel times revealed that poorly educated women chose distant hospitals less frequently than highly educated ones, thus preferring nearby facilities (*p* < 0.001). Model 3 also showed that less educated women chose more frequently hospitals delivering lower volumes of interventions (*p* = 0.029). Furthermore, Model 3 suggested that women with more comorbidities were more likely to choose hospitals with shorter average length of stay (*p* = 0.002). In general, the largest effect was observed for interactions between educational level and travel distance and between number of comorbidities and hospital stay. Finally, the effects of age and nationality were never significant.

As shown in Table 3, the mean travel time elasticity for the three Models was -1.36%, -1.31%, and -0.77%, respectively. Focusing on Model 3, it means that the chance of choosing a hospital would decrease by 0.77% for a 1% increase in the natural logarithm of travel times. For instance, considering a 20-min trip [Ln (travel times) = 3.0)], the chance of choosing that hospital would fall by 15.5% for a 20% increase in Ln (travel times), from 3.0 to 3.6—which means a 37-min travel. On the contrary, we computed the mean waiting time elasticity only for Model 1 (− 0.27%) because the effect of waiting times in Model 2 and Model 3 was not significant.

**Table 3:** Average elasticities of demand for travel times and waiting times (expressed as percentages)

Elasticities				
	Ln (travel times)		Waiting times	
	Mean	SD	Mean	SD
Model 1	− 1.36	0.87	− 0.27	0.24
Model 2	− 1.31	0.88	−	−
Model 3	− 0.77	− 0.60	−	−

Elasticity for waiting times in Models 2 and 3 was not computed as the variable was not statistically significant

## Discussion

In this paper, we applied mixed logit regression models to the hospital choice for POP surgery. We investigated the relationship between the hospital choice and the patient’s willingness to travel and to wait, considering at the same time other quality- and efficiency-related hospital characteristics and the women’s available demographic data. We found that travel times more than waiting times influenced between-hospital patient choices. Proximity (shorter travel times) was the main determinant of the hospital choice, as confirmed by regression models in which travel time absolute coefficients were the highest. The average elasticity was higher for travel than for waiting times, supporting previous results.

Interaction analysis showed that poorly educated women were less likely to choose distant hospitals and hospitals providing a greater total amount of interventions compared to their counterpart. Taking the educational level as a proxy of the socioeconomic status, this evidence suggests that the hospital choice of women with potential financial trouble might have been restricted by limited travel means. The difficulty in accessing health services by vulnerable groups raises concerns over vertical equity—intended as the allocation of different resources for different needs. On the other hand, women with multiple comorbidities preferred hospitals with shorter lengths of stay, suggesting that women with a more complex clinical profile sought to choose the most efficient hospital possible to receive surgery. Since the previous literature suggests that robotic and laparoscopic interventions ensure shorter length of stay, the perception of hospital efficiency may be related to the provision of minimally invasive surgery, which could be a determinant of hospital choice [55, 56].

These findings are in line with the literature. Several studies have demonstrated that the main determinants of the hospital choice are distances and waiting times [21, 47, 57, 58]. For instance, we found that mean travel times were 19.1 (± 16.6) minutes. A previous study from New Mexico, USA, investigating the determinants of patient choice for conservative *vs.* surgical management showed that patients choosing POP surgery had a geometric travel mean of 19.5 miles [59]. Furthermore, we found that socioeconomic conditions widely influenced the hospital choice, with a significant pro-rich inequity in the access to health services, as previously shown [60–64].

Possible solutions to vertical inequality may be improved public and patient transportation to mitigate the effect of distance, a more uniform implementation and diffusion of new technologies (e.g., robotic surgical systems) that make hospitals perceived as being of better quality, and availability of nearby accommodations affiliated with the hospital. In addition, inequity may benefit from an improved planning and organizational capacity at the point of service, which may consist of a more uniform booking system for elective surgery at the regional level. Finally, the empowerment of telemedicine and teleconsultation for presurgical evaluation could reduce the impact of travel distance.

The main limits of this work are related to the data quality and availability. First, possible gaps in the harmonization of the various booking systems for elective surgery may affect the waiting time measurement from regional health databases, leading to an underestimation of the effect. Second, administrative databases lack information on some patient characteristics, such as income brackets, family conditions, lifestyle, the employment status, and the place of residence. In fact, unlike clinical registries, administrative data do not allow the exact clinical profile of patients to be captured due to their intrinsic nature. This limitation was partly overcome by the use of mixed logit models, which allowed specifying individual random coefficients, thus accounting for the heterogeneity of unobserved preferences [54]. Third, potential coding error might have affected the correct identification of the surgical approach used for each woman (e.g., robotic surgery). In addition, details on the specific surgical technique used for each patient (e.g., sacrocolpopexy) and on the POP stage (according to the POP quantification system) were missing. As another limitation, our findings are not generalizable at the national level since our work was carried out in a single region.

Furthermore, we could not establish if patients were aware of possible alternative providers in choosing hospitals for surgery; so, we just knew where patients were operated on, ignoring whether their choice was taken actively or not [15]. In addition, we could not determine to which extent recommendations by general practitioners and private specialists influenced patient choice. Finally, we explore just between-hospital patient choice for POP surgery, and not patient choice for conservative *vs.* surgical treatment [59], as we lacked individually collected data from the entire Tuscan female population.

Therefore, we propose to perform further studies to explore individual preferences and better define the decision-making process by patients and physicians. For this purpose, the main determinants of both patient choice and physicians’ prescriptive behaviors could be intercepted through Discrete Choice Experiments (DCEs). DCEs are quasi-experimental analyses in which surveys can be administered to patients or physicians after a randomization process to make them choose between different hypothetical scenarios according to several attributes identified from the literature to be the main factors influencing their choices [42, 65].

Despite these limits, this is the first paper—to our knowledge—investigating between-hospital patient choice for POP surgery in Italy. Moreover, while previous studies on patient choice for POP surgery were performed (outside the Italian context) by employing survey data [65, 66], we used real-world administrative health data, which have been largely adopted by health policymakers and managers to assess hospital performance [67]. Health administrative data of Tuscany are well-validated and reliable sources as their quality is routinely checked by the Regional Health Information System Office. Very similar data sources and methodologies were previously employed by Seghieri et al*.* [21]. We tried to apply the same validated study design to a different clinical context, replicating data management and analysis methods.

Furthermore, this paper shows the existence of large variation in treatment rates for POP in Tuscany, providing an insight into some determinants of unwarranted variation [68]. As a matter of fact, travel distances were the main determinants of the hospital choice for POP surgery; and the health districts with no provider hospitals or with hospitals providing few interventions—from which there was greater mobility—had among the lowest treatment rates in Tuscany. We also observed a negative, albeit weak, correlation between mobility and treatment rates. Given that women—particularly, less educated women—tend to travel short distances to receive POP surgery, the need to travel and move for receiving surgery (related to the lack of supply) could discourage women, especially less educated ones, from undergoing POP surgery. This could result in an undertreatment that might not be justified by the real differences in women’s needs. In any case, our study can only suggest this conclusion, not having sufficient inferential statistical power to prove it with certainty.

## Conclusions

We found that the between-hospital choice of patients requiring major POP surgery in Tuscany was influenced mainly by travel times, and partially by waiting times. Less educated women preferred hospitals nearby and hospitals providing lower volumes of interventions compared to women with higher education, while women with comorbidities received surgery in more efficient hospitals. These findings stress the importance of exploring and tracking health equity, particularly vertical equity—which is related to the identification of population subgroups with peculiar needs—, in order to improve, quality, efficiency and accessibility of health services [21, 69]. Managers and policymakers should consider how patients behave in choosing providers to mitigate patients’ unmet needs and equity gaps [62]. Finally, the information asymmetry between health providers and users seems to be accentuated for the weaker socioeconomic groups, potentially suggesting the failure of quasi-market policies based on the assumption of rational choices taken by well-informed individuals [70].

### Supplementary Information

Below is the link to the electronic supplementary material.

Supplementary file1 (DOCX 115 KB)

Supplementary file2 (PNG 122 KB)

Supplementary file3 (JPG 646 KB)

Supplementary file4 (PNG 124 KB)

## Data Availability

The authors declare that the manuscript is an honest, accurate, and transparent account of the study being reported; that no important aspects of the study have been omitted; and that any discrepancies from the study as originally planned have been explained. Aggregated data and data management procedures that support the findings of this study are available upon reasonable request. However, individual-level data are not available for privacy reasons. Similarly, the Stata file-do that contains the procedure for running mixed logit models cannot be shared because its intellectual property belongs to statistics professor Chiara Seghieri.

## References

[1] Ferre F, Belvis D, Iuli AG, Valerio L, Longhi S, Lazzari A, Fattore G, Ricciardi W, Maresso A (2014). Italy: health system review. Health. Syst. Transit..

[2] France G, Taroni F (2005). The evolution of health-policy making in Italy. J. Heal. Polit. Policy. Law..

[3] Fattore G, Torbica A (2006). Inpatient reimbursement system in Italy: how do tariffs relate to costs?. Health. Care. Manag. Sci..

[4] Brekke KR, Canta C, Siciliani L, Rune O (2021). Hospital competition in a national health service: evidence from a patient choice reform. J. Health Econ..

[5] Beckert W, Christensen M, Collyer K (2012). Choice of NHS-funded hospital services in England. Econ. J..

[6] Cooper Z, Gibbons S, Jones S, Mcguire A (2011). Does hospital competition save lives? Evidence from the English NHS patient choice reforms. Econ. J..

[7] Coulter, A.: Do patients want a choice and does it work? BMJ. 341, (2010) 10.1136/bmj.c4989 20947576

[8] Bevan G, Evans A, Nuti S (2019). Reputations count: Why benchmarking performance is improving health care across the world. Heal. Econ. Policy. Law..

[9] Berkowitz E, Flexner W (1981). The market for health care services: is there a non-traditional consumer?. J. Heal. Care. Mark..

[10] Lane P, Lindquist J (1988). Hospital choice: a summary of the key empirical and hypothetical findings of the 1980s. Mark. Health. Serv..

[11] Gaynor M, Propper C, Seiler S (2016). Free to choose? Reform, choice, and consideration sets in the english national health service. Am. Econ. Rev..

[12] Pilkington H, Blondel B, Drewniak N, Zeitlin J (2012). Choice in maternity care: associations with unit supply, geographic accessibility and user characteristics. Int. J. Health Geogr..

[13] Bauer J, Klingelhöfer D, Maier W, Schwettmann L, Groneberg D (2020). Prediction of hospital visits for the general inpatient care using floating catchment area methods: a reconceptualization of spatial accessibility. Int. J. Health. Geogr..

[14] Balia, S., Brau, R., Marrocu, E.: What drives patient mobility across Italian regions? Evidence from hospital discharge data. Heal. Care Provis. Patient Mobil. 133–154 (2014) 10.1007/978-88-470-5480-6_6 24864385

[15] Aggarwal A, Lewis D, Mason M, Sullivan R, Van Der Meulen J (2017). Patient mobility for elective secondary health care services in response to patient choice policies: a systematic review. Med. Care Res. Rev..

[16] Exworthy M, Peckham S (2006). Access, choice and travel: implications for health policy. Soc. Policy Adm..

[17] Nuti, S., Vainieri, M.: Strategies and tools to manage variation in regional governance systems. (2014)

[18] Propper C, Damiani M, Leckie G, Dixon J (2007). Impact of patients’ socioeconomic status on the distance travelled for hospital admission in the English National Health Service. J. Heal. Serv. Res. Policy..

[19] Cook PA, Downing J, Wheater CP, Bellis MA, Tocque K, Syed Q, Phillips-Howard PA (2009). Influence of socio-demographic factors on distances travelled to access HIV services: enhanced surveillance of HIV patients in north west England. BMC. Pub. Health..

[20] Varkevisser M, van der Geest SA, Schut FT (2012). Do patients choose hospitals with high quality ratings? Empirical evidence from the market for angioplasty in the Netherlands. J. Health Econ..

[21] Seghieri C, Calovi M, Ferrè F (2018). Proximity and waiting times in choice models for outpatient cardiological visits in Italy. PLoS ONE.

[22] Sivey P (2012). The effect of waiting time and distance on hospital choice for English cataract patients. Health Econ..

[23] Giannini A, Russo E, Malacarne E, Cecchi E, Mannella P, Simoncini T (2019). Role of robotic surgery on pelvic floor reconstruction. Minerva. Ginecol..

[24] De Gouveia De Sa, M., Claydon, L.S., Whitlow, B., Dolcet Artahona, M.A.: Robotic versus laparoscopic sacrocolpopexy for treatment of prolapse of the apical segment of the vagina: a systematic review and meta-analysis. Int. Urogynecol. J. 27: 355–366 (2016). 10.1007/s00192-015-2763-0 10.1007/s00192-015-2763-0 26249235

[25] Performance Evaluation System of Sant’Anna School of Advanced Studies, https://performance.santannapisa.it/pes/start/start.php

[26] Nuti S, Vola F, Bonini A, Vainieri M (2014). Making governance work in the health care sector: evidence from a “natural experiment” in Italy. Heal. Econ. Policy Law..

[27] Nuti S, Seghieri C, Vainieri M (2013). Assessing the effectiveness of a performance evaluation system in the public health care sector: Some novel evidence from the Tuscany region experience. J. Manag. Gov..

[28] Tuscany Regional Health Agency: Gynaecologic surgery for benign diseases in Tuscany [La chirurgia ginecologica per patologia benigna in Toscana]. (2018)

[29] Mannella P, Giannini A, Russo E, Naldini G, Simoncini T (2015). Personalizing pelvic floor reconstructive surgery in aging women. Maturitas.

[30] Giannini A, Caretto M, Russo E, Mannella P, Simoncini T (2019). Advances in surgical strategies for prolapse. Climacteric.

[31] Jelovsek JE, Maher C, Barber MD (2007). Pelvic organ prolapse. Lancet.

[32] Coolen A-LWM, van Oudheusden AMJ, van Eijndhoven HWF, van der Heijden TPFM, Stokmans RA, Mol BWJ, Bongers MY (2013). A comparison of complications between open abdominal sacrocolpopexy and laparoscopic sacrocolpopexy for the treatment of vault prolapse. Obstet. Gynecol. Int..

[33] Hudson CO, Northington GM, Lyles RH, Karp DR (2014). Outcomes of robotic sacrocolpopexy: a systematic review and meta-analysis. Fem. Pelv. Med. Reconstr. Surg..

[34] Mereu L, Tateo S, D’Alterio MN, Russo E, Giannini A, Mannella P, Pertile R, Cai T, Simoncini T (2020). Laparoscopic lateral suspension with mesh for apical and anterior pelvic organ prolapse: a prospective double center study. Eur. J. Obstet. Gynecol. Reprod. Biol..

[35] Ferrari A, Manetti S, Giannini A, Simoncini T, Vainieri M (2021). PSU14 Assessing unwarranted variation in minimally-invasive surgery for pelvic organ prolapse in Tuscany, Italy: a case study (Conference Abstract). Value. Heal..

[36] Nuti S, Seghieri C (2014). Is variation management included in regional healthcare governance systems Some proposals from Italy. Health. Pol. (N. Y.).

[37] Lungu DA, Foresi E, Belardi P, Nuti S, Giannini A, Simoncini T (2021). The impact of new surgical techniques on geographical unwarranted variation: the case of benign hysterectomy. Int. J. Environ. Res. Public Health..

[38] Wennberg JE (2011). Time to tackle unwarranted variations in practice. BMJ.

[39] Mulley, A., Trimble, C., Elwyn, G.: Patients’ preferences matter: stop the silent misdiagnosis. King’s Fund. 1–64 (2012) 10.1136/bmj.e6572 23137819

[40] Ward MM (2021). Regional variation in surgical procedure rates: going beyond description. JAMA. Surg..

[41] Cafagna G, Seghieri C, Vainieri M, Nuti S (2018). A turnaround strategy: Improving equity in order to achieve quality of care and financial sustainability in Italy. Int. J. Equity Health..

[42] De Bekker-Grob E, Ryan M, Gerard K (2012). Discrete choice experiments in health economics: a review of the literature. Health Econ..

[43] Italian Data Protection Authority: General authorisation to process personal data for scientific research purposes, https://www.garanteprivacy.it/home/docweb/-/docweb-display/docweb/1884019#

[44] Calovi M, Seghieri C (2018). Using a GIS to support the spatial reorganization of outpatient care services delivery in Italy. BMC Health Serv. Res..

[45] Austin SR, Wong YN, Uzzo RG, Beck JR, Egleston BL (2015). Why summary comorbidity measures such as the Charlson Comorbidity Index and Elixhauser score work. Med. Care..

[46] Van Walraven C, Austin PC, Jennings A, Quan H, Forster AJ (2009). A modification of the Elixhauser comorbidity measures into a point system for hospital death using administrative data. Med. Care..

[47] Gutacker N, Siciliani L, Moscelli G, Gravelle H (2016). Choice of hospital: which type of quality matters?. J. Health Econ..

[48] Kulinskaya E, Kornbrot D, Gao H (2005). Length of stay as a performance indicator: robust statistical methodology. IMA J. Manag. Math..

[49] Aplin B, Nazzal M, Qu W, Ph MDD, Zelenock G, Kazan V, Abbas J (2015). Patient variables impacting hospital costs from 2008 to 2010. Am. J. Surg..

[50] Phillips KA, Harold SL (1997). The policy implications of using hospital and physician volumes as “indicators” of quality of care in a changing health care environment. Int. J. Qual. Heal. Care..

[51] LaPar DJ, Kron IL, Jones DR, Stukenborg GJ, Kozower BD (2012). Hospital procedure volume should not be used as a measure of surgical quality. Ann. Surg..

[52] Walther F, Kuester D, Bieber A, Malzahn J, Rüdiger M, Schmitt J (2021). Are birth outcomes in low risk birth cohorts related to hospital birth volumes? A systematic review. BMC. Preg. Childbirth..

[53] Mahmoudi E, Lu Y, Chang SC, Lin CY, Wang YC, Chang CJ, Cheng MH, Chung KC (2017). Associations of surgeon and hospital volumes with outcome for free tissue transfer by using the national Taiwan population health care data from 2001 to 2012. Plast. Reconstr. Surg..

[54] Lippi Bruni M, Ugolini C, Verzulli R (2021). Should I wait or should I go? Travelling versus waiting for better healthcare. Reg. Sci. Urban Econ..

[55] Li H, Sammon J, Roghmann F, Sood A, Ehlert M (2014). Utilization and perioperative outcomes of robotic vaginal vault suspension compared to abdominal or vaginal approaches for pelvic organ prolapse. Can. Urol. Assoc. J..

[56] Geller E, Siddiqui N, Wu J, Visco A (2008). Short-term outcomes of robotic sacrocolpopexy compared with abdominal. Obstet. Gynecol..

[57] Avdic D, Moscelli G, Pilny A, Sriubaite I (2019). Subjective and objective quality and choice of hospital: evidence from maternal care services in Germany. J. Health. Econ..

[58] Kuklinski D, Vogel J, Geissler A (2021). The impact of quality on hospital choice Which information affects patients’ behavior for colorectal resection or knee replacement?. Health. Care. Manag. Sci..

[59] Dunivan GC, Fairchild PS, Cichowski SB, Rogers RG (2014). The association between distances traveled for care and treatment choices for pelvic floor disorders in a rural southwestern population. J. Health Dispar. Res. Pract..

[60] Moscelli G, Siciliani L, Gutacker N, Cookson R (2018). Socioeconomic inequality of access to healthcare: does choice explain the gradient?. J. Health Econ..

[61] Smith H, Currie C, Chaiwuttisak P, Kyprianou A (2018). Patient choice modelling: how do patients choose their hospitals?. Health Care Manag. Sci..

[62] Barsanti S, Nuti S (2014). The equity lens in the health care performance evaluation system. Int. J. Health Plann. Manage..

[63] Masseria C, Giannoni M (2010). Equity in access to health care in Italy: a disease-based approach. Eur. J. Public Health..

[64] Glorioso V, Subramanian SV (2014). Equity in access to health care services in Italy. Health Serv. Res..

[65] Notten KJB, Essers BA, Weemhoff M, Rutten AGH, Donners JJAE, Van Gestel I, Kruitwagen RFPM, Roovers JPWR, Dirksen CD (2015). Do patients prefer mesh or anterior colporrhaphy for primary correction of anterior vaginal wall prolapse: a labelled discrete choice experiment. BJOG An Int. J. Obstet. Gynaecol..

[66] Kapoor DS, Thakar R, Sultan AH, Oliver R (2009). Conservative versus surgical management of prolapse: what dictates patient choice?. Int. Urogynecol. J..

[67] Dhruva SS, Ross JS, Desai NR (2018). Real-world evidence: promise and peril for medical product evaluation. P T..

[68] Lungu DA, Grillo Ruggieri T, Nuti S (2019). Decision making tools for managing waiting times and treatment rates in elective surgery. BMC Health Serv. Res..

[69] Akinci F, Esatoglu A, Tengilimoglu D, Parsons A (2004). Hospital choice factors: a case study in Turkey. Health Mark. Q..

[70] Fotaki M (2007). Patient choice in healthcare in England and Sweden: from quasi-market and back to market? A comparative analysis of failure in unlearning. Public Adm..

