# Supplementary material for: Driving time drives the hospital choice: choice models for pelvic organ prolapse surgery in Italy
Source: Eur J Health Econ. 2023 Jan 11;24(9):1575–86. doi: 10.1007/s10198-022-01563-6 (PMC9833017; doi:10.1007/s10198-022-01563-6)
Supplement: Supplementary file 1 — Supplementary file1 (DOCX 115 KB) [file 10198_2022_1563_MOESM1_ESM.docx]

**SUPPLEMENTARY INFORMATION**


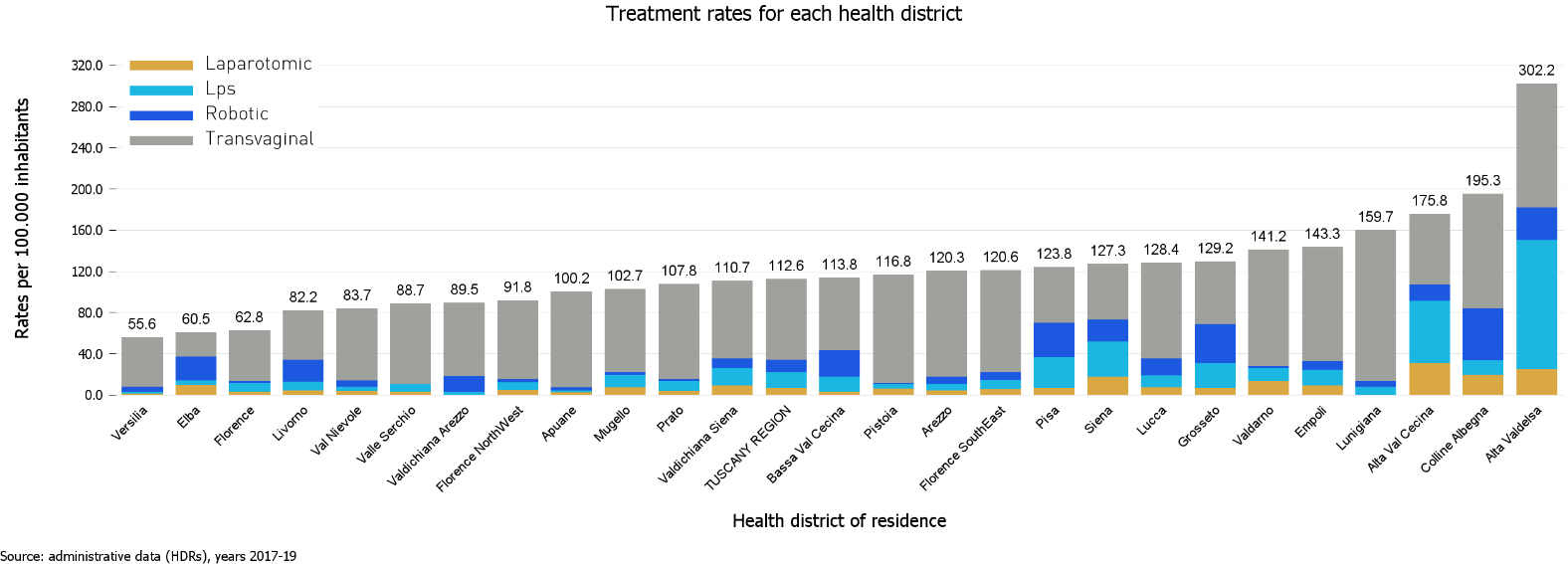


**Fig S3.** Three-year treatment rates for POP surgery in the Tuscany Region and in each health district from 2017 to 2019 (per 100,000 women). Treatment rates were computed by dividing the number of POP interventions for the 2017-2019 female population residing in each health district regardless of where the intervention was provided. Total treatment rates were also split into the specific treatment rates for each surgical procedure delivered

| 1. **ICD9-CM codes for pelvic organ prolapse surgery** | |
| --- | --- |
| Diagnosis codes | 618.00, 618.09, 618.1, 618.2, 618.3, 618.4, 618.5, 618.01 |
| Procedure codes | 69.22, 70.77, 68.31, 68.39, 68.59, 59.79, 00.39, 71.79, 70.50, 70.51, 70.52 |
| Exclusion criteria 1 | Transvaginal anterior/posterior colporrhaphy (procedure codes: 70.50, 70.51, 70.52) with no concomitant hysterectomy (procedure codes: 68.31, 68.39, 68.41, 68.49, 68.51, 68.59, 68.61, 68.69, 68.71, 68.79, 68.9) |
| Exclusion criteria 2 | Major Diagnosis Category 14 (“Pregnancy, Childbirth, and Puerperium”) and cancer or trauma (diagnosis codes: 179, 180.0, 180.1, 180.8, 180.9, 181, 182.0, 182.1, 182.8, 183.0, 183.2, 183.3, 183.4, 183.5, 183.8, 183.9, 184.0, 184.1, 184.2, 184.3, 184.4, 184.8, 184.9, 869.0, 869.1, 233.1, 233.2, 233.3, 236.0, 236.1, 236.2, 236.3, 867.4, 867.5, 867.6, 867.7, 867.8, 867.9, 868.00, 868.03, 868.04, 868.09, 868.10, 868.13, 868.14, 868.19, 879.6, 879.7, 879.8, 879.9, 906.0, 908.1, 908.2, 939.1, 947.4, 233.0, 233.1, 233.2, 233.9) |
|  |  |
| 1. **ICD9-CM codes for each surgical approach** | |
| Robot-assisted surgery | 00.39 |
| Laparoscopic surgery | 54.21, 68.31, 68.41, 68.61 |
| Transvaginal surgery | 68.51, 68.59, 68.71, 68.79, 70.50, 70.51, 70.52 |
| Open laparotomy surgery | 69.22, 70.77 (when other codes absent) |

**Table S4**. Algorithm for cohort selection from Hospital Discharge Records: A) ICD9-CM diagnosis and procedure codes used to identify women hospitalized for receiving pelvic organ prolapse surgery, with the specification of exclusion criteria; B) ICD9-CM procedure codes used to identify the surgical approach performed on each woman

| **Patients** | **Hospital where the patient was operated** | **Provider hospitals** | **Median waiting times for each hospital** | **Ln (travel times)** | **Choice** |
| --- | --- | --- | --- | --- | --- |
| Patient #1 | Hospital 2 | Hospital 1 | 67,5 | 3,70 | 0 |
| Patient #1 | Hospital 2 | Hospital 2 | 180 | 2,47 | **1** |
| Patient #1 | Hospital 2 | Hospital 3 | 206 | 3,09 | 0 |
| Patient #1 | Hospital 2 | Hospital 4 | 107 | 2,85 | 0 |
| Patient #1 | Hospital 2 | Hospital 5 | 177 | 3,37 | 0 |
| Patient #1 | Hospital 2 | Hospital 6 | 88 | 3,29 | 0 |
| Patient #1 | Hospital 2 | Hospital 7 | 202,5 | 3,92 | 0 |
| Patient #1 | Hospital 2 | Hospital 8 | 137 | 4,44 | 0 |
| Patient #1 | Hospital 2 | Hospital 9 | 195 | 4,30 | 0 |
| Patient #1 | Hospital 2 | Hospital 10 | 26,5 | 4,94 | 0 |
| Patient #1 | Hospital 2 | Hospital 11 | 105 | 4,71 | 0 |
| Patient #1 | Hospital 2 | Hospital 12 | 200 | 4,00 | 0 |
| Patient #1 | Hospital 2 | Hospital 13 | 225 | 3,77 | 0 |
| Patient #1 | Hospital 2 | Hospital 14 | 202 | 3,76 | 0 |
| Patient #1 | Hospital 2 | Hospital 15 | 124 | 4,17 | 0 |
| Patient #1 | Hospital 2 | Hospital 16 | 171 | 4,51 | 0 |
| Patient #1 | Hospital 2 | Hospital 17 | 145 | 3,27 | 0 |
| Patient #1 | Hospital 2 | Hospital 18 | 93 | 4,11 | 0 |
| Patient #1 | Hospital 2 | Hospital 19 | 176 | 3,53 | 0 |
| Patient #1 | Hospital 2 | Hospital 20 | 67 | 3,29 | 0 |
| Patient #1 | Hospital 2 | Hospital 21 | 36 | 4,36 | 0 |
| Patient #1 | Hospital 2 | Hospital 22 | 195 | 3,84 | 0 |
| Patient #2 | Hospital 8 | Hospital 1 | 67,5 | 4,78 | 0 |
| Patient #2 | Hospital 8 | Hospital 2 | 180 | 4,57 | 0 |
| Patient #2 | Hospital 8 | Hospital 3 | 206 | 4,28 | 0 |
| Patient #2 | Hospital 8 | Hospital 4 | 107 | 4,45 | 0 |
| Patient #2 | Hospital 8 | Hospital 5 | 177 | 4,24 | 0 |
| Patient #2 | Hospital 8 | Hospital 6 | 88 | 4,49 | 0 |
| Patient #2 | Hospital 8 | Hospital 7 | 202,5 | 4,75 | 0 |
| Patient #2 | Hospital 8 | Hospital 8 | 137 | 0,08 | **1** |
| Patient #2 | Hospital 8 | Hospital 9 | 195 | 3,43 | 0 |
| Patient #2 | Hospital 8 | Hospital 10 | 26,5 | 5,03 | 0 |
| Patient #2 | Hospital 8 | Hospital 11 | 105 | 4,83 | 0 |
| Patient #2 | Hospital 8 | Hospital 12 | 200 | 4,01 | 0 |
| Patient #2 | Hospital 8 | Hospital 13 | 225 | 3,86 | 0 |
| Patient #2 | Hospital 8 | Hospital 14 | 202 | 3,97 | 0 |
| Patient #2 | Hospital 8 | Hospital 15 | 124 | 4,34 | 0 |
| Patient #2 | Hospital 8 | Hospital 16 | 171 | 4,10 | 0 |
| Patient #2 | Hospital 8 | Hospital 17 | 145 | 4,65 | 0 |
| Patient #2 | Hospital 8 | Hospital 18 | 93 | 3,74 | 0 |
| Patient #2 | Hospital 8 | Hospital 19 | 176 | 4,27 | 0 |
| Patient #2 | Hospital 8 | Hospital 20 | 67 | 4,60 | 0 |
| Patient #2 | Hospital 8 | Hospital 21 | 36 | 4,28 | 0 |
| Patient #2 | Hospital 8 | Hospital 22 | 195 | 3,88 | 0 |
| We show here two patients (Patient #1 and Patient #2) operated in 2017 in two different hospitals (Hospital 2 and Hospital 8). When the hospital where the patient received surgery coincided with one of the 22 provider hospitals of the list, the variable "*choice*" took value 1. Median waiting times (as well as length of stay and volumes) were calculated at the hospital level, so they were the same for each patient-hospital combination. In contrast, the travel times (expressed as a natural logarithms) that each patient would spend to reach, from her residence, each of the 22 hospitals of the list were different from patient to patient and therefore varied for each hospital-patient combination. The "*rand*" option used in the "*mixlogit*" command on Stata allowed us to specify in our models that the coefficients of Ln (Travel Times) varied randomly for each patient-hospital combination. | | | | | |

**Table S5.** Example of the construction of the database.
