# Supplementary figures and images for: Driving time drives the hospital choice: choice models for pelvic organ prolapse surgery in Italy
Source: Eur J Health Econ. 2023 Jan 11;24(9):1575–86. doi: 10.1007/s10198-022-01563-6 (PMC9833017; doi:10.1007/s10198-022-01563-6)

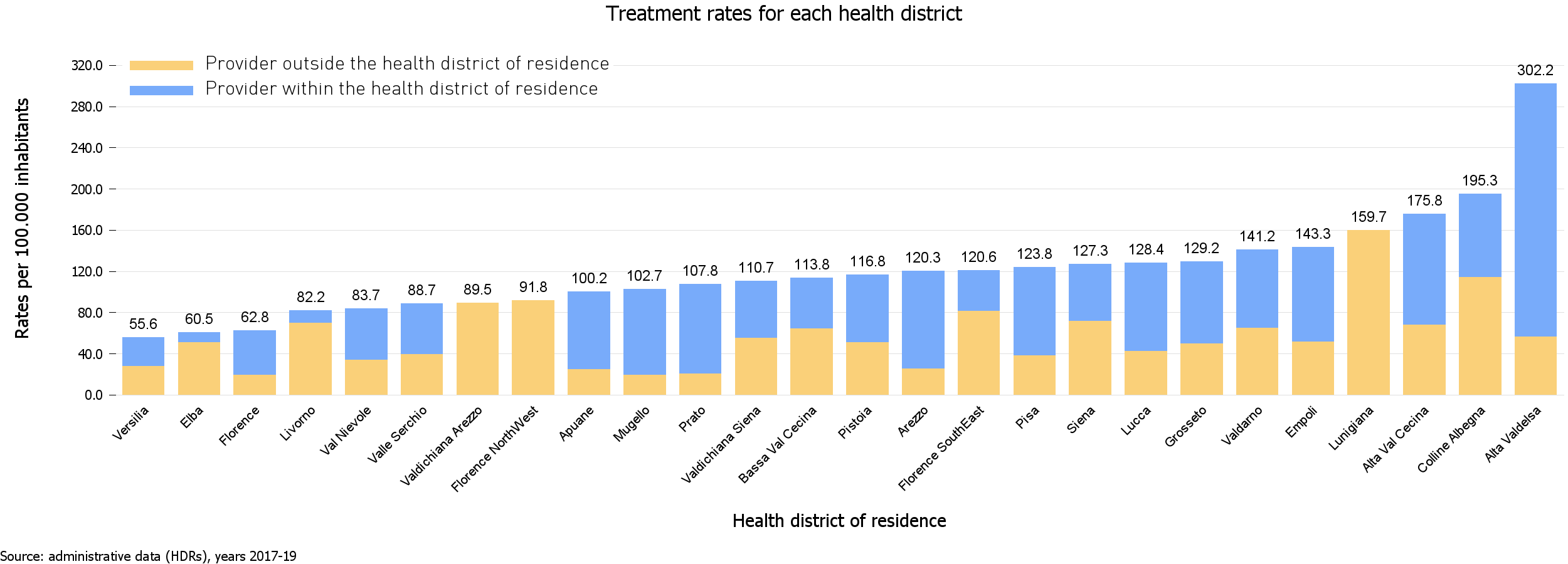

Supplement: Supplementary file 2 — Supplementary file2 (PNG 122 KB) [file 10198_2022_1563_MOESM2_ESM.png]

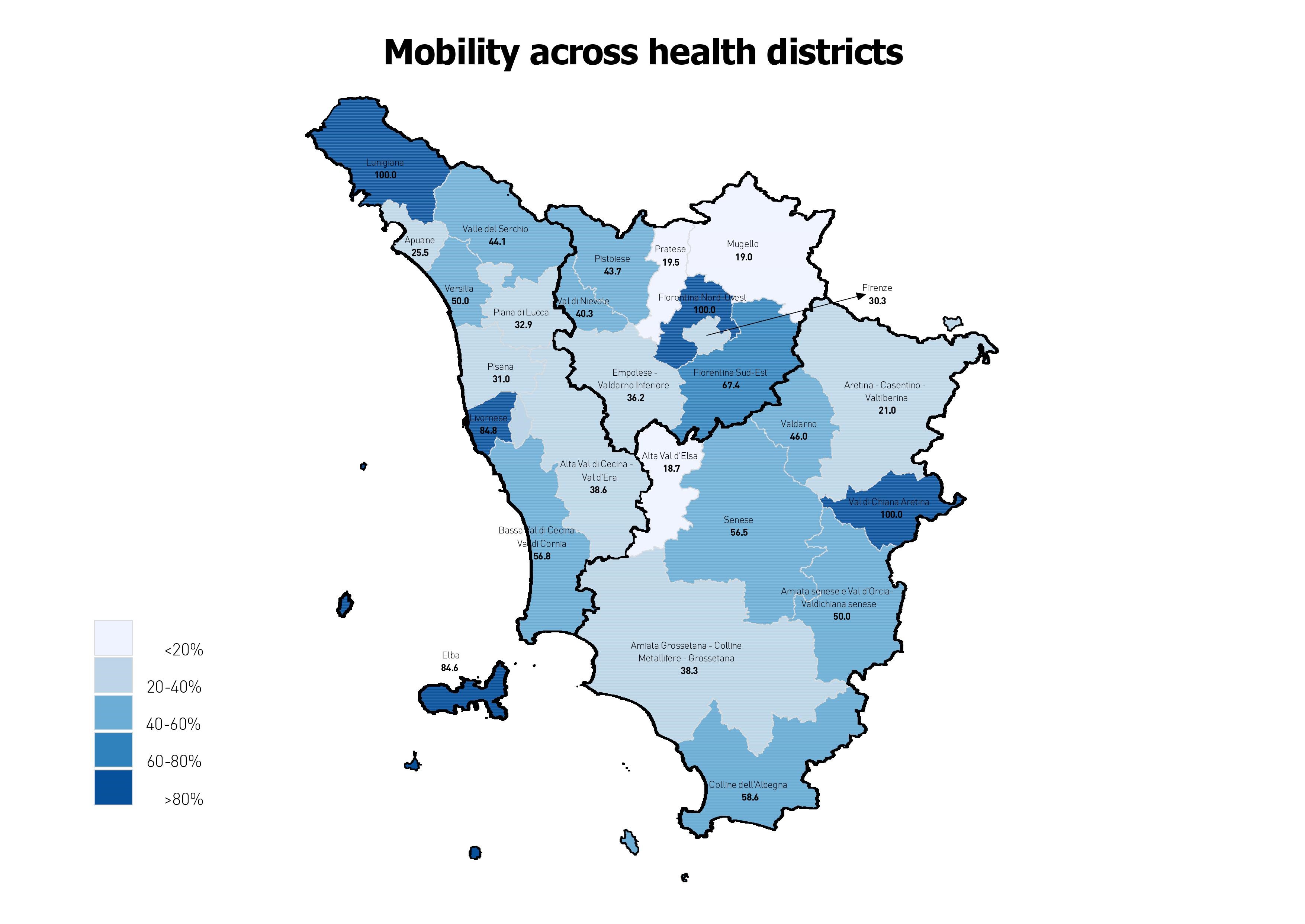

Supplement: Supplementary file 3 — Supplementary file3 (JPG 646 KB) [file 10198_2022_1563_MOESM3_ESM.jpg]

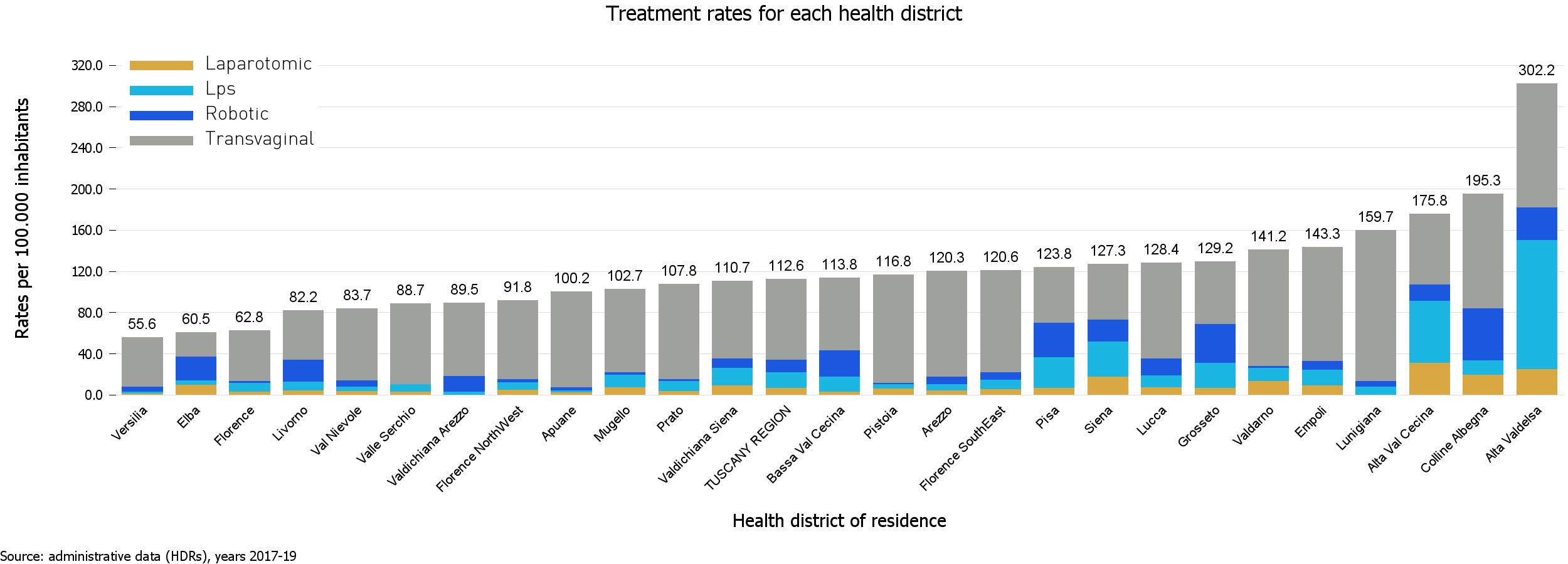

Supplement: Supplementary file 4 — Supplementary file4 (PNG 124 KB) [file 10198_2022_1563_MOESM4_ESM.png]
